# Lazertinib in *EGFR*-Variant Non–Small Cell Lung Cancer With CNS Failure to Prior EGFR Tyrosine Kinase Inhibitors: A Nonrandomized Controlled Trial

**DOI:** 10.1001/jamaoncol.2024.2640

**Published:** 2024-08-15

**Authors:** Min Hee Hong, Yoon Ji Choi, Hee Kyung Ahn, Sun Min Lim, Bhumsuk Keam, Dong-Wan Kim, Tae Min Kim, Jeonghwan Youk, Yu Jung Kim, Shinwon Hwang, Sangwoo Kim, Ju Won Kim, Hye Ryun Kim, Jin Hyoung Kang

**Affiliations:** 1 Division of Medical Oncology, Department of Internal Medicine, Yonsei Cancer Center, Severance Hospital, Yonsei University College of Medicine, Seoul, Republic of Korea; 2 Division of Medical Oncology and Hematology, Department of Internal Medicine, Korea University Anam Hospital, Korea University College of Medicine, Seoul, Republic of Korea; 3 Division of Medical Oncology, Department of Internal Medicine, Gachon University Gil Medical Center, Incheon, Republic of Korea; 4 Department of Internal Medicine, Seoul National University Hospital, Seoul, Republic of Korea; 5 Department of Internal Medicine, Seoul National University Bundang Hospital, Seoul National University College of Medicine, Seongnam, Republic of Korea; 6 Department of Biomedical Systems Informatics, Yonsei University College of Medicine, Seoul, Republic of Korea; 7 Department of Biomedical Systems Informatics and Graduate School of Medical Science, Yonsei University College of Medicine, Seoul, Republic of Korea; 8 Division of Medical Oncology, Department of Internal Medicine, Seoul St Mary’s Hospital, College of Medicine, The Catholic University of Korea, Seoul, Republic of Korea; 9 Department of Medicine, Physician-Scientist Program, Yonsei University College of Medicine, Seoul, Republic of Korea

## Abstract

**Importance:**

*EGFR*-variant non–small cell lung cancer (NSCLC) is associated with a high rate of central nervous system (CNS) metastases, even with treatment with first-generation or second-generation epidermal growth factor receptor (EGFR) tyrosine kinase inhibitors (TKIs).

**Objective:**

To investigate CNS activity with lazertinib, a third-generation EGFR TKI.

**Design, Setting, and Participants:**

This multicenter single-arm, phase 2 nonrandomized controlled trial was conducted in South Korea and included patients with *EGFR*-variant NSCLC who had asymptomatic or mildly symptomatic brain metastases after unsuccessful treatment with first-generation or second-generation EGFR TKIs. Data were collected from June 2021 to April 2022, with a data cutoff date of December 15, 2022.

**Exposure:**

Lazertinib, 240 mg, once daily.

**Main Outcomes and Measures:**

The primary end point was intracranial objective response rate (iORR) in the evaluable population according to the Response Evaluation Criteria in Solid Tumours version 1.1 assessed by the investigators. Secondary end points included intracranial progression-free survival (iPFS) and iORR in patients with T790M-negative disease and isolated CNS progression as well as overall ORR, duration of response, intracranial duration of response, disease control rate, overall survival, cerebrospinal fluid penetration of lazertinib, and safety.

**Results:**

Among 40 included patients, 25 (63%) were women, and the median (range) age was 63 (29-85) years. A total of 38 patients were evaluable for tumor response, including 12 patients with leptomeningeal metastases. At data cutoff, the median (range) follow-up was 13.6 (2.9-17.7) months. The iORR for the evaluable population was 55% (21 of 38; 95% CI, 38.3-71.4); for patients with T790M-positive disease, 80% (4 of 5; 95% CI, 28.4-99.5); for patients with T790M-negative disease, 43% (9 of 21; 95% CI, 21.8-66.0); and for patients with T790M-unknown disease, 67% (8 of 12; 95% CI, 34.9-90.1). The median iPFS was 15.8 months (95% CI, 15.2-not reached) for the evaluable population, 15.2 months (95% CI, 4.2-not reached) for the T790M-positive subgroup, 15.4 months (95% CI, 7.9-not reached) for the T790M-negative subgroup, and 18.0 months (95% CI, 3.9-not reached) for the T790M-unknown subgroup. The cerebrospinal fluid penetration rate of lazertinib was 46.2% (95% CI, 10.0-49.6), providing further support for its mechanism of intracranial response. Most adverse events were grade 1 or 2.

**Conclusions and Relevance:**

In this study, lazertinib had substantial CNS activity, regardless of T790M status, against the progression of intracranial metastases with or without leptomeningeal metastases after unsuccessful treatment with first-generation or second-generation EGFR TKIs in patients with metastatic *EGFR*-variant NSCLC. These results suggest that using lazertinib instead of brain local treatment could be a potential strategy in patients with *EGFR*-variant NSCLC whose CNS metastases progressed after prior EGFR TKI treatment.

**Trial Registration:**

ClinicalTrials.gov Identifier: NCT05326425

## Key Points

**Question:**

What is the central nervous system (CNS) activity of lazertinib, a third-generation epidermal growth factor receptor (EGFR) tyrosine kinase inhibitors (TKI), in patients with *EGFR*-variant non–small cell lung cancer (NSCLC) who have brain metastases after unsuccessful treatment with first-generation or second-generation EGFR TKIs?

**Findings:**

In this single-arm, phase 2 nonrandomized controlled trial involving 40 patients who experienced CNS progression with first-generation or second-generation EGFT TKI, the intracranial objective response rate was 55% (21 of 38 patients). The median intracranial progression-free survival was 15.8 months.

**Meaning:**

In this study, lazertinib showed substantial CNS activity in treating intracranial metastases in patients with *EGFR*-variant NSCLC, suggesting it could be an alternative to brain local treatment for those who progressed after prior EGFR TKIs.

## Teaser

This nonrandomized controlled trial investigates central nervous system (CNS) activity with lazertinib, a third-generation epidermal growth factor receptor (EGFR) tyrosine kinase inhibitors.

## Introduction

*EGFR*-variant non–small cell lung cancer (NSCLC) responds effectively to epidermal growth factor receptor (EGFR) tyrosine kinase inhibitors (TKIs).^1^ In some cases, treatment with a third-generation EGFR TKI, such as osimertinib, or a first-generation or second-generation EGFR TKI, like gefitinib, erlotinib, or afatinib, is the standard therapy for advanced NSCLC harboring activating *EGFR* variants, while osimertinib is a preferred option based on the superior brain activity.^2,3^

Central nervous system (CNS) metastases commonly occur in patients with *EGFR*-variant NSCLC.^4^ The CNS is a sanctuary site for metastases due to the presence of an active blood-brain barrier (BBB). Approximately 25% of patients with newly diagnosed advanced *EGFR*-variant NSCLC already have CNS metastases at diagnosis, and half of patients develop brain metastases throughout the disease course. CNS metastases are a well-known adverse prognostic factor for overall survival (OS) in patients with lung cancer.^5,6^ Therefore, particularly for patients with *EGFR*-variant NSCLC, controlling CNS metastases is a crucial factor in determining their treatment approach.^7^ Thus, the properties of drugs that can effectively penetrate the BBB are highly important for the successful management of brain metastases.^8^

First-generation or second-generation EGFR TKIs show a limited response to CNS metastases, while third-generation EGFR TKIs, known for better BBB penetration, have a favorable intracranial response.^5,9,10^ Lazertinib is a third-generation EGFR TKI that targets T790M as well as sensitizing variants, sparing wild-type *EGFR* and demonstrating highly active CNS activity.^11^ It is only approved in the Republic of Korea for treatment in patients with *EGFR*-variant T790M-positive locally advanced or metastatic NSCLC previously treated with EGFR TKI or as first-line treatment for patients with metastatic NSCLC whose tumors have *EGFR* exon 19 deletion or L858R variants.^11^ As lazertinib is not a substrate for the efflux transporters, such as the breast cancer resistance protein, and shows weak affinity for P-glycoprotein, it is anticipated to be minimally affected by efflux transporters at the BBB.^11^ Preclinical studies in an *EGFR*-variant brain metastases mouse model demonstrated that lazertinib effectively crosses the BBB, leading to inhibition of intracranial tumor growth and indicating its potential for CNS efficacy.^12^ In a recent phase 3 clinical study (LASER301) comparing lazertinib with gefitinib, lazertinib demonstrated a significantly longer survival outcome, with a median progression-free survival (PFS) of 20.6 months and a hazard ratio of 0.45, indicating its efficacy as a first-line treatment in patients with *EGFR*-variant NSCLC.^13^ In the brain metastases subgroup of the LASER301 study, lazertinib extended intracranial PFS (iPFS), achieving more durable responses compared with gefitinib.^14^

However, currently, use of a third-generation EGFR TKI is limited as a primary treatment, with subsequent use restricted to patients with T790M-variant disease.^11^ Therefore, investigating the efficacy of treatments with excellent BBB permeability to intracranial tumors, regardless of T790M status, is important in patients with *EGFR*-variant NSCLC with brain metastases.

We conducted a phase 2 single-arm nonrandomized controlled trial of lazertinib in patients with *EGFR*-variant NSCLC with CNS metastases following disease progression after prior first-generation or second-generation EGFR TKIs to evaluate CNS activity, irrespective of presence of the T790M variant.

## Methods

### Trial Design and Patient Selection

This was a single-arm, phase 2 nonrandomized controlled trial conducted in Korea involving patients with *EGFR*-variant metastatic NSCLC with asymptomatic or mildly symptomatic brain metastases after failure of first-generation or second-generation EGFR TKIs. The patients received lazertinib, 240 mg, once daily, with the primary end point being the intracranial objective response rate (iORR) among the evaluable population, which was defined as individuals who had undergone an evaluation of their tumor response following their initial baseline assessment. Secondary end points included iPFS, ORR, duration of response (DoR), disease control rate (DCR), OS, among others. The study was conducted in compliance with the protocol (Supplement 1), general ethical standards (such as the Declaration of Helsinki), and all relevant laws and regulations. The study protocol was approved by the institutional review boards of each participating institute and the Severance Institutional Review Board, and all recruited patients provided written informed consent. This study followed the Consolidated Standards of Reporting Trials (CONSORT) reporting guideline.

Key inclusion criteria were as follows: First, age 20 years or older with an Eastern Cooperative Oncology Group performance status of 0 to 2 and histologically or cytologically confirmed advanced and/or metastatic NSCLC. Second, treatment failure of first-generation or second-generation EGFR TKIs, including gefitinib, afatinib, and erlotinib; a single course of cytotoxic chemotherapy was permitted. Third, presence of *EGFR* 19del or L858R before treatment with EGFR TKIs. Fourth, asymptomatic or mildly symptomatic brain metastases that did not require steroid therapy. The distinction between asymptomatic or mildly symptomatic brain metastases was based on comprehensive clinical evaluations and history taking. Asymptomatic or mildly symptomatic leptomeningeal metastases (LM) were permitted. Fifth, confirmed T790M variant status in tissue or blood after EGFR TKI failure. Sixth, measurable intracranial metastases with a maximum diameter of 10 mm or more on computed tomography or magnetic resonance imaging scans (previously radiated lesions were not permitted, but they could be allowed if there was evidence of progression three months after radiation). Key exclusion criteria were as follows: First, exposure to third-generation EGFR TKIs. Second, 2 or more courses of cytotoxic chemotherapy. Data were collected from June 2021 to April 2022, with a data cutoff of December 15, 2022.

### Treatment Plan

Patients were given lazertinib, 240 mg, once daily regardless of their meals. Each treatment cycle lasted for 42 days, and tumor response according to Response Evaluation Criteria in Solid Tumours (RECIST) version 1.1 was assessed after each cycle for the first 4 evaluations. Subsequently, evaluations were conducted every 2 cycles starting from the fifth evaluation onwards. Treatment was continued until withdrawal of consent, inability to participate in the study, serious protocol noncompliance, or disease progression. However, in the case of disease progression, treatment could be continued if there was clinical benefit per the physician’s discretion. Dose reduction to lazertinib, 160 mg, was allowed if patients experienced adverse drug reactions during the administration of lazertinib, 240 mg. All eligible patients provided blood samples for blood-based next-generation sequencing (NGS) using Guardant360 CDx (Guardant Health) both prior to the first dose of lazertinib and after disease progression after lazertinib treatment.

### Study End Points

The primary end point was iORR among the evaluable population. The set of patients evaluable for response was defined as those who underwent a postbaseline tumor response evaluation. iORR was defined as the percentage of patients with intracranial complete response (CR) or partial response (PR) according to the investigator assessed RECIST version 1.1. Secondary end points were iPFS, iORR in patients with T790M-negative disease and isolated CNS progression, overall ORR, DoR, intracranial DoR (iDoR), DCR, OS, treatment failure pattern (intracranial progression, extracranial progression, or both), and salvage intracranial treatment rate. iPFS was calculated from the initiation of treatment to the first documented intracranial disease progression or death from any cause, whichever occurred first. iDoR was measured from the first treatment response (either CR or PR) to the intracranial disease progression or death due to any cause, whichever came earlier. Exploratory end points included (1) baseline genetic variant status and iORR, (2) changes in genetic variants in plasma samples at baseline and disease progression through liquid biopsy NGS analysis, and (3) lazertinib drug concentration in cerebrospinal fluid (CSF).

### Statistical Analysis

A difference of 45% vs an iORR of 25%, which is based on the historical control from the pemetrexed-platinum arm in the AURA3 trial, at a 1-tailed significance level of .05, with a power of 0.8, required an exact single-stage phase 2 design with 36 patients. Assuming a 10% dropout rate, a total of 40 patients were needed. Variables expressed as ratios (iORR, ORR, and DCR) presented the number and percentage of patients along with 95% CIs on both sides. Variables expressed as time (iPFS, PFS, OS, and DoR) were represented using Kaplan-Meier graphs and median survival time. All analyses were conducted using SAS version 9.4 (SAS Institute).

## Results

### Demographic Characteristics

**Figure 1.**
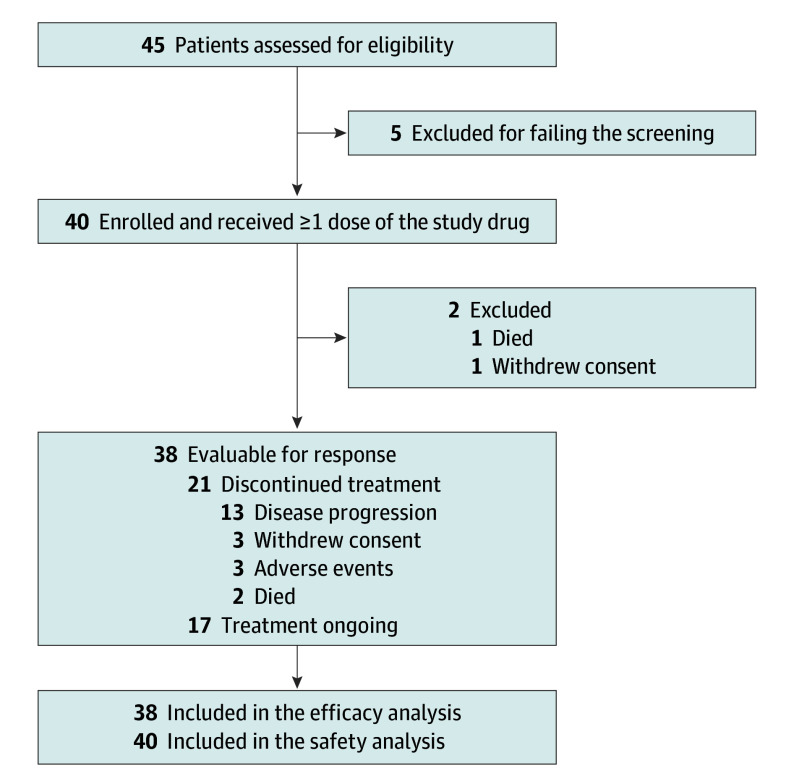
CONSORT Diagram Two patients discontinued treatment (1 death and 1 patient decision) before the first posttreatment imaging scan; thus, they were excluded from the evaluable population.

A total of 45 patients were screened, and 40 patients were enrolled and received treatment (Figure 1). For the enrolled population, the median (range) age at diagnosis was 63 (29-85) years (Table 1). The evaluable population included 38 patients because 2 patients could not be evaluated: 1 died because of rapid tumor progression and the other refused to continue participating in the clinical trial prior to the first tumor evaluation. Half of the enrolled patients had an exon 19 deletion, while the other half had the L858R variant. All patients received a first-generation or second-generation EGFR TKIs. After failure of prior EGFR TKI treatment, tissue rebiopsy was tried in 4 patients but *EGFR* T790M was not detected. There were 5 patients with T790M variants based on blood-based NGS using Guardant360 prior to lazertinib treatment. Patients with detectable ctDNA but without T790M variants and those who had tissue biopsies confirming T790M negativity were collectively defined as having T790M-negative disease, comprising 23 individuals. Among the 14 patients with undetectable ctDNA, excluding the 2 who were confirmed as having T790M-negative disease through tissue rebiopsy, the remaining 12 were classified as having T790M-unknown disease.

**Table 1.** Baseline Characteristics

Characteristic	No. (%)			
	Total (N = 40)	T790M positive (n = 5)	T790M negative (n = 23)	Unknown T790M status (n = 12)
Age, median (range), y	63 (29-85)	62 (57-83)	66 (47-85)	56.5 (29-71)
Sex				
Female	25 (62.5)	3 (60.0)	14 (60.9)	8 (66.7)
Male	15 (37.5)	2 (40.0)	9 (39.1)	4 (33.3)
ECOG performance status				
0	6 (15.0)	1 (20.0)	2 (8.7)	3 (25.0)
1	32 (80.0)	4 (80.0)	20 (87)	8 (66.7)
2	2 (5.0)	0	1 (4.3)	1 (8.3)
Histology				
Adenocarcinoma	37 (92.5)	5 (100)	22 (95.7)	10 (83.3)
Other^a^	3 (7.5)	0	1 (4.3)	2 (16.7)
Smoking history				
Never	27 (67.5)	3 (60.0)	16 (69.6)	8 (66.7)
Ever smoker	13 (32.5)	2 (40.0)	7 (30.4)	4 (33.3)
*EGFR* variants prior to previous EGFR TKIs				
Exon 19 deletion	20 (50.0)	2 (40.0)	9 (39.1)	9 (39.1)
L858R	20 (50.0)	3 (60.0)	14 (60.9)	3 (13.0)
Type of previous treatment^b^				
Afatinib	18 (45.0)	2 (40.0)	12 (52.2)	4 (33.3)
Gefitinib	18 (45.0)	3 (60.0)	8 (34.8)	7 (58.3)
Erlotinib	5 (12.5)	NA	4 (17.4)	1 (8.3)
Cytotoxic chemotherapy	10 (25.0)	1 (20.0)	9 (39.1)	NA
Isolated CNS failure^c^	9 (22.5)	2 (40.0)	6 (26.1)	1 (8.3)
Previous local CNS treatment, overall				
Stereotactic radiosurgery	12 (30.0)	1 (20.0)	7 (30.4)	4 (33.3)
Whole-brain radiation	5 (12.5)	NA	4 (17.4)	1 (8.3)
Leptomeningeal carcinomatosis				
Based on brain MRI imaging	12 (30.0)	NA	7 (30.4)	5 (41.7)
Confirmed by CSF	2 (5.0)	NA	NA	2 (16.7)
Symptom severity groups for brain metastases				
Asymptomatic	30 (75.0)	5 (100)	16 (69.6)	9 (75.0)
Mildly symptomatic	10 (25.0)	NA	7 (30.4)	3 (25.0)

Abbreviations: CNS, central nervous system; CSF, cerebrospinal fluid; ECOG, Eastern Cooperative Oncology Group; EGFR, epidermal growth factor receptor; MRI, magnetic resonance imaging; NA, not applicable; TKI, tyrosine kinase inhibitors.

^a^ The other category included 2 non–small cell lung cancer, not otherwise specified and 1 squamous cell carcinoma.

^b^ One patient received both gefitinib and erlotinib sequentially during the treatment course.

^c^ Isolated CNS failure refers to the progression or recurrence of cancer specifically within the CNS, which includes the brain and spinal cord.

### Efficacy

**Figure 2.**
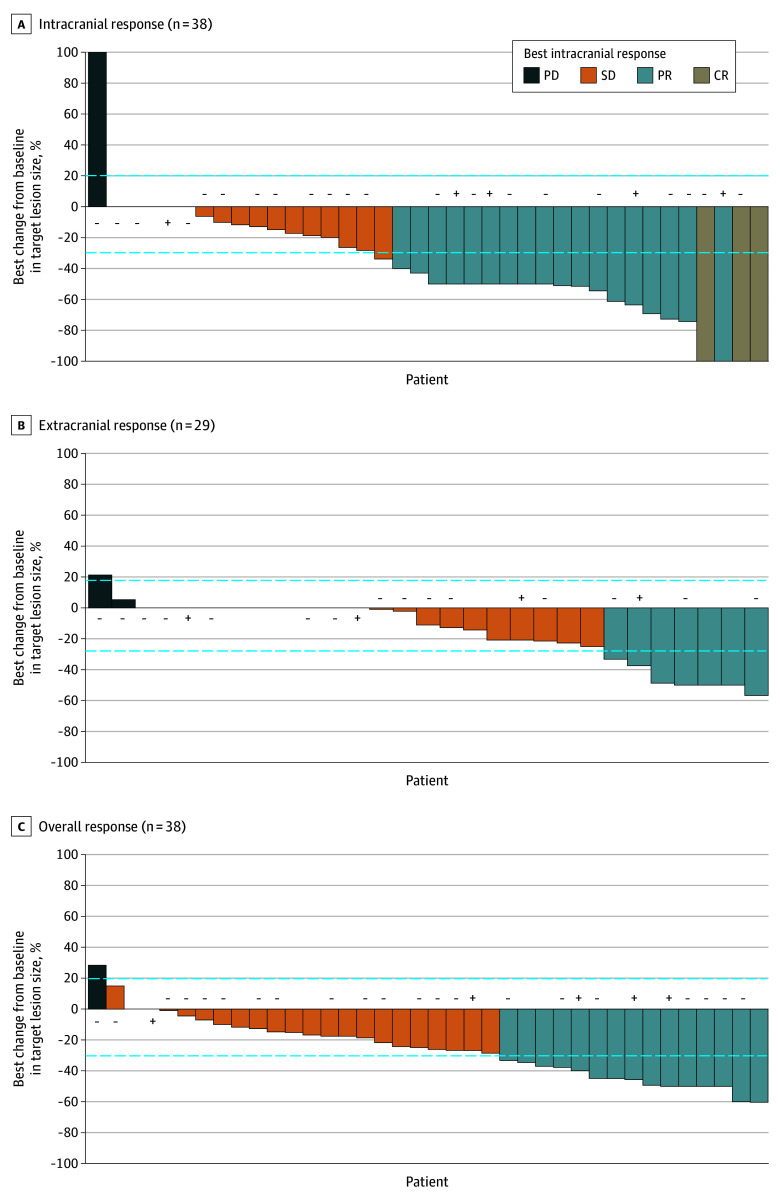
Tumor Reduction and Responses in the Evaluable Population Waterfall plot of intracranial response (A), extracranial response (B), and overall response (C). The best percentage change from baseline was calculated from the start of lazertinib treatment. Response was assessed by Response Evaluation Criteria in Solid Tumours version 1.1 per investigator review. Bars with a + sign indicate patients with T790M-positive disease; a − sign, T790M-negative disease; and without any sign, T790M-unknown disease. CR indicates complete response; PD, progressive disease; PR, partial response; SD, stable disease.

For intracranial response, an iORR of 55% (21 of 38; 95% CI, 38.3-71.4) was achieved with confirmed intracranial CR observed in 3 patients and intracranial PR seen in 18 patients (Table 2; Figure 2A). Among patients with T790M-positive disease, intracranial PR was detected in 4 patients, resulting in an iORR of 80% (4 of 5; 95% CI, 28.4-99.5). In those with T790M-negative disease, 2 achieved intracranial CR and 7 PR, giving an iORR of 43% (9 of 21; 95% CI, 21.8-66.0), while those with T790M-unknown disease had an iORR of 67% (8 of 12; 95% CI, 34.9-90.1). The intracranial DCR was 97% (37 of 38; 95% CI, 86.2-99.9) in all patients, 100% (5 of 5; 95% CI, 47.8-100) in those with T790M-positive disease, 95.2% (20 of 21; 95% CI, 76.2-99.9) in those with T790M-negative disease, and 100% (12 of 12; 95% CI, 73.5-100) in those with in those with T790M-unknown disease (Table 2). Intracranial tumor shrinkage was observed in most patients (Figure 2A). Based on the baseline neurologic symptom group, the median (IQR) PFS for asymptomatic patients was not reached (15.2 months to not reached), and the median (IQR) PFS was 15.4 (3.9-15.8) months in mildly symptomatic patients (eFigure 1A in Supplement 2).

**Table 2.** Intracranial, Extracranial, and Overall Response in the Evaluable Population

Population	ORR, No. (%; 95% CI)	No. (%)					
		DCR^a^	CR	PR	SD	PD	Not available
Total (N = 38)							
Overall	15 (39.5; 24.0-56.6)	37 (97.4)	NA	15 (39.5)	22 (57.9)	1 (2.6)	NA
Intracranial	21 (55.3; 38.3-71.4)	37 (97.4)	3 (7.9)	18 (47.4)	16(42.1)	1 (2.6)	NA
Extracranial	7 (18.4; 8.9-33.7)	34 (89.5)	NA	7 (18.4)	27 (71.1)	2 (5.3)	2 (5.3)
T790M positive (n = 5)							
Overall	3 (60.0; 14.7-94.7)	5 (100)	NA	3 (60.0)	2 (40.0)	NA	NA
Intracranial	4 (80.0; 28.4-99.5)	5 (100)	NA	4 (80.0)	1 (20.0)	NA	NA
Extracranial	1 (20; 2-64)	5 (100)	NA	1 (20.0)	4 (80.0)	NA	NA
T790M negative (n = 21)							
Overall	7 (33.3; 14.6-57.0)	20 (95.2)	NA	7 (33.3)	13 (61.9)	1 (4.8)	NA
Intracranial	9 (42.9; 21.8-66.0)	20 (95.2)	2 (9.5)	7 (33.3)	11 (52.4)	1 (4.8)	NA
Extracranial	3 (14.3; 4.1-35.5)	18 (85.7)	NA	3 (14.3)	15 (71.4)	2 (9.5)	1 (4.8)
Unknown T790M status (n = 12)							
Overall	5 (41.7; 15.2-72.3)	12 (100)	NA	5 (41.7)	7 (58.3)	NA	NA
Intracranial	8 (66.7; 34.9-90.1)	12 (100)	1 (8.3)	7 (58.3)	4 (33.3)	NA	NA
Extracranial	3 (25.0; 5.5-57.2)	11 (91.7)	NA	3 (25.0)	8 (66.7)	NA	1 (8.3)

Abbreviations: CR, complete response; DCR, disease control rate; NA, not applicable; ORR, objective response rate; PD, progressive disease; PR, partial response; SD, stable disease.

^a^ DCR was defined as the proportion of patients who achieved a confirmed CR, confirmed PR, or SD.

For extracranial response, extracranial ORR was 18% (7 of 38; 95% CI, 8.9-33.7) in all patients and extracranial DCR was 90% (34 of 38; 95% CI, 75.2-97.1). In patients with T790M-positive disease, extracranial ORR and DCR were 20% (1 of 5; 95% CI, 0.5-71.6) and 100% (5 of 5; 95% CI, 47.8-100), respectively (Table 2). In patients with T790M-negative disease, extracranial PR was observed in 3 patients and extracranial stable disease was identified in 15 patients, resulting in an extracranial ORR of 14% (3 of 21; 95% CI, 3.1-36.3) and extracranial DCR of 86% (18 of 21; 95% CI, 63.7-97.0). In patients with T790M-unknown disease, extracranial ORR (25% [3 of 12; 95% CI, 5.5-57.2]) and DCR (92% [11 of 12; 95% CI, 61.5-99.8]) were higher than in those with T790M-negative disease (Figure 2B).

For overall response, the ORR was 40% (15 of 38), with a confirmed PR in 15 patients. The ORR was 60% (3 of 5) among patients with T790M-positive disease and 33% (7 of 21) among those with T790M-negative disease (Table 2; Figure 2C). The DCR was 100% (5 of 5; 95% CI, 47.8-100), 95% (20 of 21; 95% CI, 76.2-99.9), and 100% (12 of 12; 95% CI, 73.5-100) for those with T790M-positive disease, T790M-negative disease, and T790M-unknown disease, respectively.

**Figure 3.**
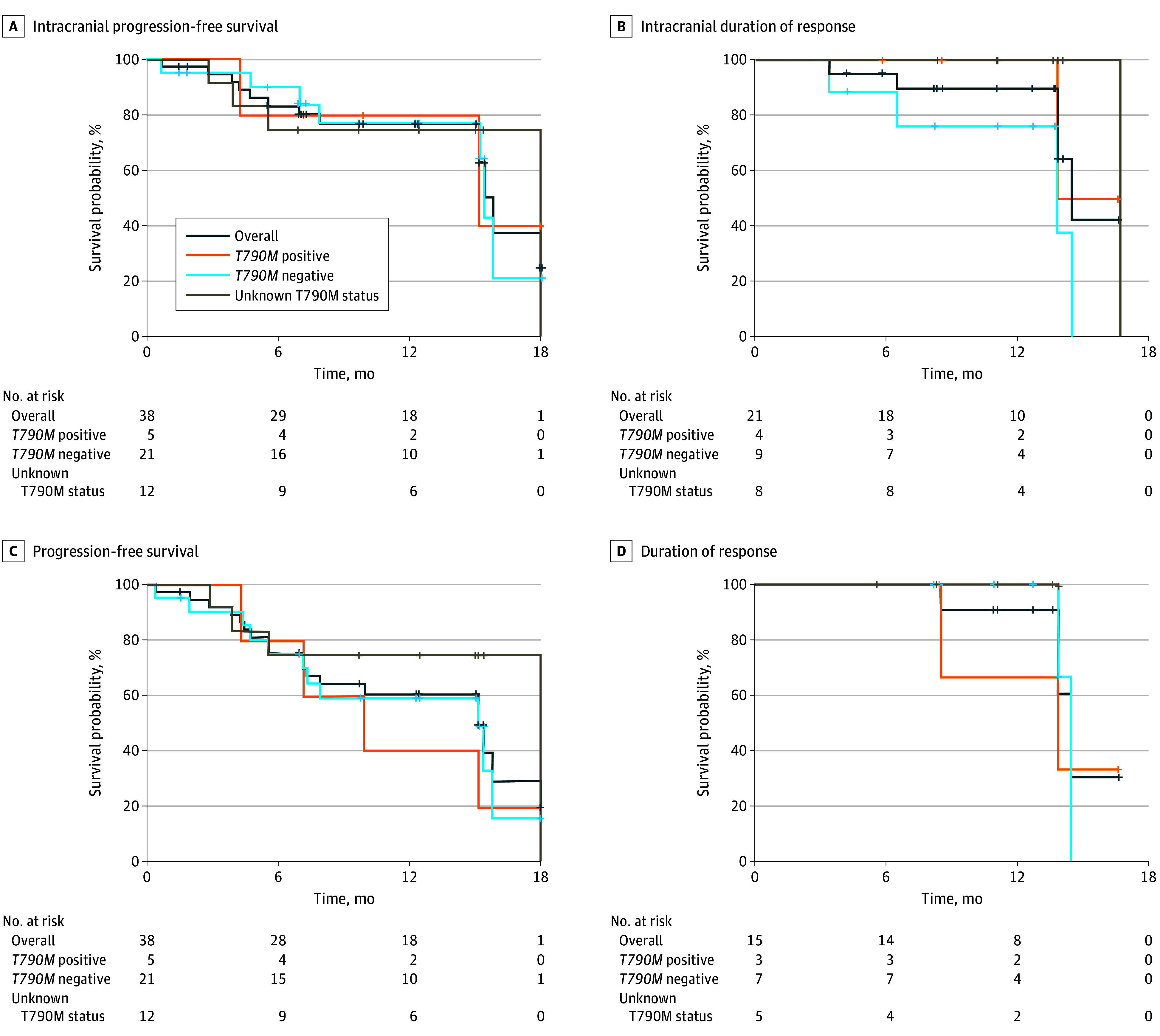
Kaplan-Meier Analyses in the Evaluable for Response Set

The median iPFS in the evaluable population, those with T790M-positive disease, those with T790M-negative disease, and those with T790M-unknown disease were 15.8 (95% CI, 15.2 to not reached), 15.2 (95% CI, 4.2 to not reached), 15.4 (95% CI, 7.9 to not reached), and 18.0 (95% CI, 3.9 to not reached) months, respectively (Figure 3A). The median iDoR in the evaluable population, those with T790M-positive disease, those with T790M-negative disease, and those with T790M-unknown disease were 14.5 (95% CI, 13.8 to not reached), not reached (95% CI, 13.8 to not reached), 13.8 (95% CI, 3.3 to not reached), and 16.7 (95% CI, not available) months, respectively (Figure 3B).

The median PFS in the evaluable population, those with T790M-positive disease, those with T790M-negative disease, and those with T790M-unknown disease was 15.2 (95% CI, 7.3-18.0), 9.9 (95% CI, 4.2 to not reached), 15.2 (95% CI, 7.1-15.8), and 18.0 (95% CI, 3.9 to not reached) months, respectively (Figure 3C). In addition, the median DoR in the evaluable population, those with T790M-positive disease, those with T790M-negative disease, and those with T790M-unknown disease was 14.5 (95% CI, 13.8 to not reached), 13.8 (95% CI, 8.5 to not reached), 14.5 (95% CI, 13.8 to not reached) months, and not reached (95% CI, not available), respectively (Figure 3D). The median OS was not reached (eFigure 1B in Supplement 2). In addition, calculations for the restricted mean iDoR and DOR were conducted, revealing a restricted mean iDoR of 13.8 months and a DoR of 13.5 months over a 17-month window period (eFigure 1C and D in Supplement 2).^15^

At the time of data cutoff and a median (IQR) follow-up of 13.6 (9.9-15.2) months, 17 patients were continuing their treatment (eFigure 2 in Supplement 2). Notably, 18 patients received lazertinib over 1 year. Time to intracranial responses was rapid, and most intracranial response was reported in 6 weeks, which was the first tumor assessment (eFigure 2A in Supplement 2). During the treatment course, 11 extracranial PD events and 9 intracranial PD events were documented (eFigure 2A and B in Supplement 2).

There were 9 patients with isolated CNS failure. Their intracranial, overall, and extracranial ORR were 78% (7 of 9; 95% CI, 40.0-97.2), 22% (2 of 9; 95% CI, 2.8-60.0), and 11% (1 of 9; 95% CI, 0.3-48.3). The intracranial and overall DCR were 100% for all subgroups (intracranial: 100% [9 of 9; 95% CI, 66.4-100]; overall: 9 of 9; 95% CI, 66.4-100), and the extracranial DCR was 78% (7 of 9; 95% CI, 40.0-97.2). Of the 9 individuals with isolated CNS failure, the median (IQR) iPFS in the evaluable population and those with T790M-positive disease were 15.2 (7.9 to not reached) months and 15.2 (15.2-15.2) months, respectively.

To verify whether lazertinib penetrates the BBB, we examined the concentrations of lazertinib and its metabolite (YH26334) in 6 paired samples of CSF and plasma. Three of 6 paired samples were collected during the fourth cycle of treatment, 2 during the sixth cycle, and 1 approximately 1 year following the initiation of treatment. These patients did not experience any dose interruptions or reductions. The median percentage of BBB penetration of lazertinib and its metabolite were 46.2% (95% CI, 26.1; 58.6) and 33.1% (95% CI, 15.1-51.5), respectively, in paired CSF and plasma samples, suggesting high CNS penetration efficacy (eTable 1 in Supplement 2).

Regarding the rescue treatment for intracranial progression, 4 patients received whole-brain radiotherapy (WBRT), while 3 underwent stereotactic radiosurgery (SRS). Among them, 2 patients in each arm took these salvage treatments during the trial.

### Safety

All patients received at least 1 dose of the study drug and were analyzed for safety. eTable 2 in Supplement 2 summarizes the overall incidence of adverse events (AEs) and treatment-related AEs (TRAEs). AEs were reported in nearly all patients (39 of 40 [98%]), and TRAEs were documented in 35 patients (88%). Fifteen patients (38%) experienced AEs of grade 3 or higher severity, and 4 (10%) were related to the treatment. Serious AEs were reported by 15 patients (38%), 5 of which (13%) were deemed treatment related. Three patients (8%) experienced AEs that led to death, but none of these events were related to the treatment. Permanent discontinuation of the treatment due to AEs was reported by 3 patients (8%).

The incidence of TRAEs is detailed in eTable 3 in Supplement 2. The most common AEs of any grade were paresthesia (18 of 40 [45%]), skin rash (17 of 40 [43%]), and pruritus (11 of 40 [28%]). Increased levels of aspartate aminotransferase and peripheral neuropathy were noted in 5 patients (13%). AEs leading to grade 3 or more were few and included diarrhea, fatigue, and pneumonitis, each observed in 1 patient (3%). Significantly, none of the most common AEs, such as paresthesia and skin rash, reached grade 3 or higher severity. It should be noted that the reported AEs were manageable, and most were of low grade.

### Genetic Variant Analysis Using Blood-Based NGS

All 40 patients underwent blood-based NGS using Guardant360 both before and after experiencing progression using lazertinib. On the initial NGS data, no somatic alterations linked to the tumor were observed in 14 patients. In addition, original *EGFR* variants, such as 19del or L858R, were detected in 16 patients. *EGFR* T790M variants were detected in 5 patients, along with other concurrent variants, such as *EGFR* amp, *PTEN*, *TP53*, and *ATM* (eFigure 3A and eTable 4 in Supplement 2). Fourteen patients underwent NGS at the time of disease progression. Of these patients, 9 (64%) were found to have at least 1 detectable acquired resistance mechanism, and 5 had detectable plasma *EGFR* variants (4 patients with *EGFR* 19del and 1 with *EGFR* L858R). Among these 5, 1 patient lost plasma *EGFR* 19del at the time of progression while using lazertinib. At the time of progression, original *EGFR* L858R or exon19 del variants were observed in 8 patients, and 4 of them did not have detectable plasma *EGFR* variants at baseline. All 3 patients with T790M present at screening had a loss of detectable plasma T790M at progression. Multiple co-occurrence variants, such as *CDK4* amp, *KRAS* amp, *FGFR3-TACC3* fusion, *TP53* variation, and *PTEN* del, were observed (eFigure 3B and eTable 4 in Supplement 2).

## Discussion

Isolated CNS progression can occur due to poor EGFR TKI penetration into the CSF.^16^ The BBB limits drug penetration to subtherapeutic doses, allowing regrowth of *EGFR*-variant disease. T790M variants may occur at a lower frequency in CNS lesions compared with the thoracic lesions in individual patients.^17^

Lazertinib showed a clinically meaningful intracranial response, with an iORR of 55% and an iPFS of 15.8 months, successfully achieving its primary end point. For overall response, an ORR of 40% and a PFS of 15.2 months were observed. In an exploratory analysis of patients with brain metastasis enrolled in the AURA3 trial, osimertinib demonstrated promising CNS efficacy in patients with T790M-positive disease, showing an iORR of 70% and an iPFS of 11.7 months.^18^ However, the CNS subset analysis of AURA3 included only those with T790M-positive disease, while most patients in the current study had T790M-negative disease.^19^ Despite the FLAURA study focusing on treatment-naive patients, its exploratory analyses reported a significant iORR of 91% and a median iPFS that was not reached with osimertinib, outperforming standard EGFR TKIs. This finding is particularly relevant to our study, which involves a predominantly T790M-negative cohort.^3^ Regarding the intracranial efficacy of osimertinib in patients with T790M-negative disease with progressive brain metastases who relapsed after EGFR TKI treatment, there are some disparities among the previous studies.^20,21,22^ Some authors supported the use of osimertinib in T790M-negative cases, while others opposed it. While the target patient population of the study is declining due to the establishment of third-generation EGFR TKIs as the standard up-front treatment, to our knowledge, this is the first published report of intracranial activity of lazertinib following prior treatment with EGFR TKIs.

The safety profile of lazertinib in this study remained consistent with findings from other previous studies, and no new AEs were identified.^23^ Most AEs were manageable at grades 1 or 2, and no grade 5 toxic effects were reported.

Notably, LM have a detrimental prognosis, with the median OS typically ranging from 3 to 10 months from the time of diagnosis, even with treatment.^24^ Due to the limited efficacy of current treatments, like WBRT or intrathecal chemotherapy, there is a high unmet medical need for patients with this condition. In a retrospective analysis of 22 patients with LM enrolled in the AURA3 trial, osimertinib showed an LM ORR of 55% and a median LM PFS of 11.1 months.^25^ In the current study, 12 patients with LM showed an iORR of 63.6% and an iPFS of 15.2 months.

Local resection, WBRT, and SRS have historically been used in the management of brain metastases.^5^ SRS could result in severe complications, such as radiation necrosis.^26^ Comparing the addition of WBRT with SRS alone, WBRT improves local control within the CNS but carries a higher risk of neurocognitive decline and does not enhance survival rates.^27^ In this context, it is suggested that for NSCLC with *EGFR* variants, the use of CNS-penetrant TKIs might allow the deferral of local brain therapy without adversely impacting disease progression.^28^ Our promising outcomes further support the potential role of lazertinib as an effective therapeutic option for patients with CNS progression after first-generation or second-generation EGFR TKIs. Despite a small number of patients, the observed higher CSF penetration rate of lazertinib compared with previous studies provides robust evidence supporting its mechanism of action in eliciting intracranial responses.^29,30^ These results indicate that in some cases, attempting lazertinib therapy instead of up-front brain local treatment could potentially become a viable treatment option for patients who have experienced failure with first-generation or second-generation EGFR TKIs and developed brain progression.

When we observed the response rates, the disparity between the extracranial ORR (18%) and the iORR (55%) to lazertinib was noteworthy. Potential factors contributing to this discrepancy could be the low number of confirmed patients with T790M-positive disease (n = 5) and the limited number of patients with extracranial target lesions (n = 29) vs intracranial target lesions (n = 38). Only 4 of 40 patients underwent tumor rebiopsy, indicating the significant limitations and constraints associated with performing tumor rebiopsies in real-world clinical practice.^31^ Whereas it is well-known that approximately half of the acquired resistance to first-generation and second-generation EGFR TKIs is caused by *EGFR* T790M variants, in the current study, only 13% of patients (n = 5) had confirmed T790M variants as an acquired resistance mechanism.^1^ The low T790M detection rate in the current study is mostly attributed to the limited frequency of rebiopsies. Considering more than half of the patients (21 of 38 [55%]) had T790M-negative disease and more than 20% of patients had isolated CNS failure prior to the study, most intracranial resistance mechanisms to the first-generation or second-generation EGFR TKIs might result from pharmacokinetic resistance (ie, failure of drug delivery to the target) rather than biological resistance, such as T790M variants. Meanwhile, extracranial resistance mechanisms may primarily derive from biological resistance. Also, this is in line with the study’s inclusion criteria and consistent with previous reports.^17,32^ Thus, our study suggested that pharmacokinetic resistance can be overcome by the relevant and highly CNS-penetrant next-generation drug without local therapy in the absence of information on T790M status. However, the low extracranial ORR (20%) in those with T90M-positive disease was not fully explained. Further research with larger sample sizes and more comprehensive molecular profiling is needed to elucidate the discrepancy.

We obtained blood-based NGS of 14 patients after initiation of lazertinib. Similar to the previous literature, all T790M amino acids present at baseline were undetectable at the time of disease progression while taking lazertinib.^23^ Genetic alterations related to resistance, including 1 case of *FGFR3-TACC3* fusion and 1 case of *KRAS* amplification, were initially identified in patients with lazertinib resistance. Other frequently detected genetic alterations in third-generation EGFR TKI resistance, such as *EGFR* C797X and *MET* amplification, were not reported.^23,33^ This may be an issue related to the small sample size, and further research is needed regarding the resistance mechanism to lazertinib. In understanding the broader implications of our study, it is essential to consider the international landscape of lazertinib’s approval and clinical application. The drug’s use in various countries provides a context for its emerging role in the treatment of *EGFR*-variant NSCLC. Furthermore, recent advances from the MARIPOSA and MARIPOSA-2 trials, exploring the combination of amivantamab and lazertinib, are reshaping our understanding of effective treatment strategies.^34,35^

### Limitations

This study has limitations. The low detection rate of T790M variants is mainly due to the infrequent occurrence of rebiopsies. The relatively small sample size, particularly in subgroups of patients with T790M-positive disease, may affect the generalizability of results and the power to detect differences between subgroups. In addition, the study’s single-arm design limits our ability to directly compare lazertinib’s efficacy with other treatments or standard of care. A key constraint of the study lies in its focus on patients with CNS progression after first-generation or second-generation EGFR TKI therapy, as this group is becoming less representative of the current patient population. As the use of upfront third-generation EGFR TKIs as first-line treatment becomes standard, the study’s findings may have limited applicability to the growing population of patients initially treated with third-generation EGFR TKIs. Additionally, the short follow-up period limits our ability to assess long-term outcomes and overall survival.

## Conclusions 

In conclusion, in this nonrandomized controlled trial, lazertinib demonstrated substantial CNS activity regardless of the presence of T790M variants, providing an effective approach against the progression of intracranial metastases in patients with *EGFR*-variant NSCLC who progressed after failure of first-generation or second-generation EGFR TKIs.

## Notes

Supplement 1. Trial Protocol

Supplement 2. **eTable 1.** Cerebrospinal Fluid (CSF) Penetration Rate of Lazertinib and Its Metabolite (YH26334) **eTable 2.** Overall Summary of Adverse Events (AEs) and Treatment-Related AEs (TRAEs) **eTable 3.** Incidence of Treatment-Related Adverse Events (TRAEs) **eTable 4.** Genetic Variant Analysis Based on Blood-Based Next-Generation Sequencing at Baseline and the Time of Progression **eFigure 1.** Intracranial PFS (iPFS) Based on the Neurologic Symptom, Overall Survival, and Restricted Mean Duration of Response (DoR) **eFigure 2.** Swimmer Plot for Treatment Duration and Time to Response of (A) Intracranial, (B) Extracranial, and (C) Overall Tumor Lesions **eFigure 3.** Genomic Alterations in (A) Baseline Samples of All Enrolled Patients (N = 40) and (B) Paired Pre- and Post-Lazertinib Samples in 14 Patients Who Showed Disease Progression by Blood-Based Next-Generation Sequencing (NGS)

Supplement 3. Data Sharing Statement
