# Supplementary material for: Lazertinib in EGFR-Variant Non–Small Cell Lung Cancer With CNS Failure to Prior EGFR Tyrosine Kinase Inhibitors: A Nonrandomized Controlled Trial
Source: JAMA Oncol. 2024 Aug 15;10(10):1342–51. doi: 10.1001/jamaoncol.2024.2640 (PMC11327907; doi:10.1001/jamaoncol.2024.2640)
Supplement: Supplement 1. — Trial Protocol [file jamaoncol-e242640-s001.pdf]

STUDY SYNOPSIS

Study Title

Phase II Trial of Lazertinib in Patients with Epidermal Growth Factor Receptor Sensitizing Mutation Positive, Metastatic Non-Small Cell Lung Cancer with Asymptomatic or Mild Symptomatic Brain Metastases After Failure of EGFR Tyrosine Kinase Inhibitor

Principal Investigator

Jin Hyung Kang, M.D., Ph.D

Medical oncology, Seoul St. Mary's Hospital

Hye Ryun Kim, M.D., Ph.D

Division of Medical Oncology, Department of Internal Medicine, Yonsei Cancer Center, Yonsei University College of Medicine

Study Site

(5+a) Institutions (undefined)

- Seoul St. Mary's Hospital
- Yonsei cancer center
- Korea University Anam Hospital

Study Design

A Phase II, biomarker-selected, open-label, multi-center study

Study Period

Enrollement: Jan 2021 – Dec 2021

Follow up: Jan 2022 – Dec 2023

Primary objectives

Intracranial objective response rates (iORR) (RECIST1.1)

Secondary objectives

a) intracranial progression-free survival (iPFS)
b) Intracranial objective response rates (iORR) in patients with T790M negative, isolated CNS progression
c) Overall objective response rates (ORR) (RECIST1.1)
d) Duration of Response (DoR)
e) Disease Control Rate (DCR)
f) Overall Survival (OS)
g) Treatment Failure Pattern (intracranial progression or extracranial progression or both)
h) Salvage Intracranial treatment rate (RT or surgery)
i) Toxicity and Safety

Exploratory objectives

a) Relationship between baseline gene mutation status and intracranial objective response rate
b) Changes in genetic mutations in plasma samples during baseline and disease progression through liquid biopsy NGS analysis (NGS: Guardant)
c) Lazertinib drug concentration in cerebrospinal fluid (only patients with consent)

Study Rationale

Molecular profiling in patients with newly diagnosed advanced non-small cell lung cancer (NSCLC) has become routine in clinical practice. Sensitizing epidermal growth factor receptor (EGFR) mutations are present in approximately 10–15% of Caucasian patients and 35–40% East Asian patients with NSCLC.
Currently, the first- and second-generation (gefitinib, erlotinib, afatinib) EGFR TKIs are recommended as first line therapy in EGFR-mutant NSCLC patients, and have clearly shown superior efficacy in term of progression-free survival (PFS), objective response rate (ORR) and quality of life (QoL) compared to chemotherapy.
Brain metastasis is common in NSCLC and ~30%-50% patients may develop brain metastases at some point during their disease course. There are few treatment options for brain metastases and the prognosis is still poor. Brain metastasis has become a critical issue and more novel strategies are urgently needed.
Of course, brain metastasis is often a problem during EGFR TKI treatment in EGFR mutant NSCLC. The blood-brain barrier (BBB) penetration rate of gefitnib and erlotinib is only about 1%. The response of intracranial disease of gefitnib or erlotinib is about 30%. Osimertinib, the 3rd generation TKI, has greatly improved this point, resulting in a BBB penetration rate of 16% and intracranial response rate of 64% in AURA II study.
Lazertinib (YH25448) is an oral, highly potent, mutant-selective and irreversible EGFR TKI that targets both the T790M mutation and activating EGFR mutations while sparing wild type-EGFR. In addition, nonclinical data suggest that lazertinib may be capable of crossing the BBB and potentially may offer better exposure in this anatomically protected location.
In a brain metastasis model, in which H1975 cells were implanted into brain parenchyma of nude mice, lazertinib achieved significant, complete tumor growth inhibition. Dose-dependent inhibition of phosphorylated-EGFR (p-EGFR). expression in both subcutaneous and intracranial tumor tissue by lazertinib treatment was well translated into in vivo efficacy. The subcutaneous tumor to plasma AUC0-last ratio of lazertinib was 3.0-5.1 indicating high tumor distribution. Lazertinib achieved cerebrospinal fluid (CSF) concentrations exceeding the IC50 value for p-EGFR inhibition in vitro. Lazertinib exhibited high BBB penetration and excellent therapeutic efficacy against both primary lung tumors and brain metastases in a T790M mutant NSCLC xenograft model.
Previous study reported intracranial response data in patients with advanced NSCLC after prior EGFR TKI, from a phase I/II study of Lazertinib. Intracranial disease control rate (IDCR) was 90.6% (95% CI, 83.5-97.8) and median intracranial PFS was not reached (95% CI 14.0, NR). In the brain metastasis population evaluable for response, a total of 22 patients were included; intracranial ORR was 54.5% (95% CI, 33.7-75.4). Lazertinib demonstrated clinically meaningful activity against brain metastases, considering that this study was conducted in patients who failed the first EGFR TKI treatment.
Currently, the use of EGFR TKI with good BBB permeability as a primary treatment in Korea is limited, and subsequent use is limited to patients with T790M mutations. Therefore, it is important to investigate the efficacy of EGFR TKI treatment with good BBB permeability on intracranial tumor regardless of T790M mutation in EGFR mutant NSCLC with brain metastases.
Thus, we are going to investigate the intracranial efficacy of Lazertinib in EGFR mutant NSCLC patients with brain metastasis.

Inclusion Criteria

1) Written consent
   A. Patients who voluntarily provided written informed consent prior to participation in the clinical trial
   B. Patients who voluntarily provide written informed consent for genetics and/or exploratory studies
2) Age and gender
   A. Male or female, 20 years of age or older
   B. Female patients must agree to the use of appropriate contraceptive methods and not be lactating, and for women of childbearing age, there must be evidence that the pregnancy test is negative prior to initiation of dosing, or that they are not fertile because they meet one of the following criteria at screening: box

- “Postmenopausal” women over the age of 50 and who are amenorrhea for at least 12 months

after stopping all exogenous hormone therapy

- Records of irreversible surgical infertility by hysterectomy, bilateral ovariectomy, or bilateral yolk resection, tubal ligation are not permitted
- Women under 50 years of age had amenorrhea for at least 12 months after stopping all exogenous hormone therapy, and the levels of luteinizing hormone (LH) and follicle stimulating hormone (FSH) were within the postmenopausal range of the laboratory. Is only recognized as a postmenopausal condition
- For women of childbearing age, appropriate contraception should be used up to 24 weeks after taking the last investigational drug.

C. Male patients who have not undergone vasectomy must consent to the use of a blocking contraception method, i.e., condom, and sperm supply is prohibited until 24 weeks after taking the last investigational drug

3) Target disease

A. Histologically or cytologically confirmed locally advanced or metastatic non-small cell lung cancer patients. This may occur as systemic recurrence after prior surgery for early stage disease or patients may be newly diagnosed with stage IIIB/C or IV disease.

B. Eastern Cooperative Oncology Group (ECOG) performance status (PS) of 0-2, with no deterioration in the last 2 weeks

C. Life expectancy judged by the Investigator of at least 3 months

D. Confirmed sensitizing EGFR mutation prior to administration of gefitinib, erlotinib, or afatinib (L858R, Exon 19 deletion mutations chould be confirmed as a record)

E. Failure after one regimen of EGFR TKI treatment. Past treatment history for locally advanced or metastatic NSCLC limited to one regimen of EGFR TKI treatment (gefitinib, erlotinib, or afatinib) and/or one palliative cytotoxic chemotherapy regimen.

F. Those who have been confirmed status of T790M mutations in tissues or blood after EGFR TKI failure (T790M positive or negative should be confirmed as a record)

G. Asymptomatic or mild symptomatic brain metastases progressed or newly confirmed patients

H. One or more intracranial measurable disease in accordance with Response Evaluation Criteria in Solid Tumors (RECIST v 1.1). The target lesion that has received previous local therapy should not be considered as measurable. However, new CNS lesion after more than 3 months of previous local therapy could be considered as target lesion.

Exclusion Criteria

1) The following interventional treatment

A. Prior treatment with lazertinib

B. Prior treatment with investigational drugs in other clinical trials within 30 days prior to the first administration

C. Patients who received cytotoxic chemotherapy for the treatment of advanced non-small cell lung cancer or other anticancer drugs other than EGFR TKI within 14 days prior to the first administration of the investigational drug

D. Prior local-regional therapy within 4 weeks prior to Day 1 of trial treatment (e.g., major

surgery, radiation therapy [with the exception of palliative bone-directed radiotherapy and radiotherapy administered to superficial lesions], hepatic arterial embolization, transcatheter arterial chemoembolization, chemoembolization, radiofrequency ablation, percutaneous ethanol injection, or cryoablation)

NOTE: palliative bone-directed radiotherapy should be within a limited field of radiation and for palliation only; it should be a short course, according to local institutional recommendations, and should be completed at least 7 days prior to the first administration of trial treatment

G. Patients currently receiving drugs or herbal supplements known as inhibitors or inducers of CYP3A4 or who cannot discontinue use at least 1 week prior to the first dose of lazertinib.

H. Previous anticancer treatment-related toxicities not recovered to baseline or Grade 0-1 (except alopecia)

2) Medical history and current disease

A. Symptomatic spinal cord compression (However, registration is allowed if steroid treatment is not required within at least 2 weeks before the start of administration of the investigational drug)

B. Symptomatic and unstable central nervous system (CNS) or brain metastasis requiring local treatment at screening. (Asymptomatic or mild symptomatic leptomeningeal metastasis is also permitted to be registered)

C. Symptomatic or intracranial bleeding that needs treatment

D. History of interstitial lung disease (ILD), drug-induced ILD, radiation pneumonitis which required steroid treatment, or any evidence of clinically active ILD

E. Carcinoma other than non-small cell lung cancer, if the investigator is judged to be inadequate to participate in this clinical trial due to evidence of severe or uncontrolled systemic disease, uncontrolled hypertension, or active bleeding tendency, or that it is difficult to follow this protocol. (Screening for chronic disease is not required)

F. Any of the following cardiovascular diaseases:

i. A history of congestive heart failure (CHF) of grade 3 or higher according to the New York Heart Association Classification (NYHA) or cardiac arrhythmia requiring treatment

ii. A History of unstable angina or myocardial infarction experienced within 6 months before the first administration of the investigational drug

iii. Left ventricular ejection fraction <50% on recent echocardiography or MUGA scan

G. Known human immunodeficiency virus (HIV) infection

H. Patients with refractory nausea and vomiting, chronic gastrointestinal disorders, inability to swallow the product, or undergoing enterectomy deemed to interfere with the proper absorption of lazertinib.

I. History of hypersensitivity to drugs

J. Clinically significant chronic infection or major medical or mental illness

K. Subjects with any concurrent medical condition or disease that will potentially compromise the conduct of the study at the discretion of the Investigators

L. History of allogeneic hematopoietic stem cell transplantation, history of whole blood transfusions that did not remove leukocytes within 120 days before the date of collection of

genetics specimens

3) Criteria for cardiology and clinical laboratory testing

A. Cardiac criteria in any of the following:

i. Based on the QTc value measured with an electrocardiogram (ECG) device during screening, the average of the correction of the QT interval (QTc) at rest on an electrocardiogram (ECG) measured three times> 470 msec

ii. Clinically important abnormalities of rhythm, conduction, or shape on the ECG at rest. For example, complete left block, 3rd degree cardiac block, 2nd degree cardiac block, PR interval> 250 msec

iii. Increased risk of QTc prolongation or arrhythmia, such as heart failure, hypokalemia, congenital QT prolongation syndrome, concomitant medications known to prolong QT intervals or cause Torsades de Point and any factors that increases the risk of QTc prolongation or arrhythmia such as family histories of prolonged QT syndrome or an unexplained sudden death of less than 40 years old within first-degree relatives

B. Laboratory index at baseline:

iv. Hemoglobin ≤ 9.0 g/dL (without transfusion or growth factor support in the preceding 14 days)

v. Neutrophils < 1.5 x 10^9/L

vi. Platelets < 100 x 10^9/L (without transfusion or growth factor support in the preceding 7 days)

vii. Total bilirubin > 1.5 x ULN or 3 x ULN with Gilbert syndrome (unconjugated hyperbilirubinemia) or liver metastasis

viii. Aspartate aminotransferase (AST)/alanine aminotransferase (ALT) > 2.5 x upper limit of normal (ULN) or in case of liver metastasis> 5 x ULN

ix. Renal impairment as evidenced by serum creatinine ≥ 1.5 x ULN, or calculated creatinine clearance (CrCl) < 50 mL/min by Cockroft-Gault formula (24-hour CrCl might be requested by the Investigator for confirmation, if calculated CrCl is < 60 mL/min. In such case, subjects with 24-hour CrCl < 50 mL/min should be excluded)

CrCl (mL/min) = [140 – age (year) x weight (kg)] / 72 x serum creatinine (mg/dL) {x 0.85 for female subjects}

Sample size calculation

The primary endpoint is intracranial objective response rate of lazertinib in patients with sentitizing EGFR mutation positive NSCLC with asymptomatic or mild symptomatic brain metastases

Assuming the target rate was set to 45% and a rate of 25% or below was considered futile. The null hypothesis that the true response rate is 25% will be tested against a two-sided alternative. This design yields a type I error rate of 0.05 and power of 0.8. According to exact single-stage phase II design, 36 patients will be enrolled initially and evaluated for iORRAssuming dropout rate of 10%, 40 patients will be required.

Efficacy assessment

1) Primary endpoint: **Intracranial objective response rate (iORR)**

- 1 cycle of treatment of this clinical trial is defined as continuous administration for 42 days
- Lesions that can be measurable according to RECIST 1.1 will be evaluated every 2 cycles for the 1st,

2nd, 3rd, and 4th evaluation, and every 4 cycles from the 5th evaluation

2) Secondary endpoints

- intracranial progression-free survival (iPFS)
- Intracranial objective response rates (iORR) in patients with T790M negative, isolated CNS progression
- Overall objective response rates (ORR) (RECIST1.1)
- Duration of Response (DoR)
- Disease Control Rate (DCR)
- Overall Survival (OS)
- Treatment Failure Pattern (intracranial progression or extracranial progression or both)
- Salvage Intracranial treatment rate (RT or surgery)

Analysis scheme & method

	**Screening**	**Every cycle (1 cycle: 42 days)[1]**			**Every 2 cycles[2]**	**Unscheduled visit[3]**	**EOT[4]**	**F/U 28 days[5]**	**F/U visit[6]**	**Survival F/U[7]**
**visit**	1	2	3[8]	4, 5, 6, 7	8 after					call
**Day**	-28 to -1	D1	D21	D43, 85, 127, 169	D11					
**Visit window(day)**	0	0	±3	±7(after 2nd cycle)	±7		+7	+7	±7	±7
**evaluation**										
consent[9]	X									
Consent for exploratory biomedical research	X									
Demographics	X									
Medical history	X									
Inclusion/exclusion criteria	X									
EGFR Mutation[10]	X									
Physical exam/ECOG	X	X	X	X	X	(X)	X	X		
Vital sign[11]	X	X	X	X	X	(X)	X	X		

[1] The treatment cycle is defined as 21 days to schedule procedures and evaluations. There is no scheduled break period between each cycle

[2] Visits will be held every 2 cycles until Visits 4, 5, 6, and 7 and every 4 cycles after Visit 8.

[3] If necessary, unscheduled visits may be conducted at the discretion of the investigator..

[4] All subjects will make a termination visit within 7 days of permanent discontinuation of the study drug, at which time all procedures corresponding to the termination visit will be performed. The closing visit should be made before the start of a new treatment, and the reason should be recorded in the case report.

[5] The follow-up visit on the 28th is conducted on the 28th (+7th) after the end visit.

[6] All subjects who discontinued the study drug without disease progression will continue to evaluate tumors every 3 months at the time of disease progression or until subsequent chemotherapy and will be monitored for all chemotherapy.

[7] All subjects will be monitored for survival and all chemotherapy after clinical trials every 6 weeks through telephone contact at least every 6 weeks from discontinuation of study drug until death or withdrawal of consent.

[8] Visits 3 weeks (21 days) after administration of the test drug to conduct toxicity assessment.

[9] Written informed consent of the subject must be obtained prior to any clinical trial-related procedure for the subject.

[10] EGFR Mutation status should be confirmed by past test records

[11] Vital signs (heart rate, blood pressure and body temperature and respiration rate) will be measured. Vital signs will be measured after the subject rests for 10 minutes.

Height	X									
Weight	X	X	X	X	X	(X)	X			
12-lead ECG[12]	X	X	X	X	X	(X)	X			
Ophthalmic exam[13]	X									
Hepatitis/HIV screening	X									
cytology[14]	X			X[15]						
**Latoratory[16]**										
biochemistry	X	X	X	X	X	(X)	X			
hematology	X	X	X	X	X	(X)	X			
urine	X	X	X	X	X	(X)	X			
Pregnancy test[17]	X	X		X	X		X			
PK (blood & CSF)[18]				X[19]						
**Tumor assessment[20]**										
RECIST 1.1	X			X	X	(X)	X		X	
**Blood sample collection**										
Blood samples for NGS[21]	**X**[22]						**X**			

---

[12] The 12-lead ECG test is measured 3 times at approximately 2 minute intervals at screening and once at subsequent visits.

[13] Additional tests are performed if necessary according to the judgment of the investigator

[14] For patients with leptomeningeal metastasis (LM) only

[15] Sample collection at Visit 4 and Visit 5 is mandatory, and after that, it can be performed additionally by determining whether and when to collect the sample at the discretion of the researcher.

[16] In the case of clinical laboratory tests, if the results of the screening tests conducted within 7 days prior to administration of the test drug are appropriate and there is no significant change in the clinical condition of the subject, the baseline test may be omitted. However, if there is no test result within 7 days before administration of the test drug, a baseline test should be performed. For further evaluation of subject safety, necessary clinical laboratory tests can be added and performed.

[17] At the screening, day1 of every cycle(Cycle day1) and termination visit, all women of childbearing potential must complete a urine or serum pregnancy test according to the clinical guidelines of the clinical trial site. If clinically necessary, repeat as needed during the treatment period.

[18] For patients with consent, PK blood and CSF samples will be collected 24 hours after dosing at Visit 4 (at 6 weeks) and Visit 5 (at 12 weeks), and hospitalization may be required to collect PK blood and CSF samples. PK blood and CSF samples must be collected on the day of sample collection, prior to administration of the test drug.

[19] After collection of PK blood and CSF samples at Visits 4 and 5, additional samples can be performed by determining whether and when the samples are collected at the discretion of the investigator.

[20] Subjects have not previously been irradiated and must have at least one lesion that can be accurately measured. Subjects who discontinued the test drug at the time of screening and until objective disease progression according to RECIST version 1.1 or without disease progression, every 2 cycles until visit 4, 5, 6, 7 until disease progression or subsequent chemotherapy, and every 4 from visit 8 Tumor assessment will be performed at each cycle. The baseline tumor evaluation during screening should be performed within 28 days of the first dose of the test drug, and if a biopsy was performed on the lesion with one measurable lesion, tumor evaluation should be performed at least 14 days after the biopsy date. If you have received cranial cavity radiation therapy, you must have a baseline brain CT/MRI after completing the treatment.

[21] Blood samples for NGS analysis will be collected on the same day as all scheduled or unscheduled tumor assessments, if possible.

[22] It is collected during the screening phase prior to the first drug administration.

CfDNA blood samples[23]	X[24]	X	X	X	X					
**Administration of investigational drug**										
lazertinib[25]		Orally administered once a day								
**F/U treatment**								X	X	
**Safety**										
Safety[26]	X	X		X	X	X	X	X		
Concomitant drugs	X	X		X	X	X	X	X		

Study planned dates

Enrollement: Jan 2021 – Dec 2021

Follow up: Jan 2022 – Dec 2023

Funding (specific amount is undefined)

- **K-MASTER (Cancer Precision Medicine Diagnosis and Treatment Enterprise)**
- **Yuhan**

Key references

1. Goss G, Tsai CM, Shepherd FA, Ahn MJ, Bazhenova L, Crinò L, de Marinis F, Felip E, Morabito A, Hodge R, Cantarini M, Johnson M, Mitsudomi T, Jänne PA, Yang JC. CNS response to osimertinib in patients with T790M-positive advanced NSCLC: pooled data from two phase II trials. Ann Oncol. 2018 Mar 1;29(3):687-693. doi: 10.1093/annonc/mdx820. PMID: 29293889.
2. Yun J, Hong MH, Kim SY, Park CW, Kim S, Yun MR, Kang HN, Pyo KH, Lee SS, Koh JS, Song HJ, Kim DK, Lee YS, Oh SW, Choi S, Kim HR, Cho BC. YH25448, an Irreversible EGFR-TKI with Potent Intracranial Activity in EGFR Mutant Non-Small Cell Lung Cancer. Clin Cancer Res. 2019 Apr 15;25(8):2575-2587. doi: 10.1158/1078-0432.CCR-18-2906. Epub 2019 Jan 22. PMID: 30670498.
3. Ahn MJ, Han JY, Lee KH, Kim SW, Kim DW, Lee YG, Cho EK, Kim JH, Lee GW, Lee JS, Min YJ, Kim JS, Lee SS, Kim HR, Hong MH, Ahn JS, Sun JM, Kim HT, Lee DH, Kim S, Cho BC. Lazertinib in patients with EGFR mutation-positive advanced non-small-cell lung cancer: results from the dose escalation and dose expansion parts of a first-in-human, open-label, multicentre, phase 1-2 study. Lancet Oncol. 2019 Dec;20(12):1681-1690. doi: 10.1016/S1470-2045(19)30504-2. Epub 2019 Oct 3. Erratum in: Lancet Oncol. 2020 Feb;21(2):e70. PMID: 31587882.

[23] Blood samples for cfDNA analysis will be collected on the same day as all scheduled or unscheduled tumor assessments, if possible.

[24] It is collected during the screening phase prior to the first drug administration.

[25] All screening procedures and clinical laboratory results must be obtained and reviewed before the subject receives the first dose of the study drug.

[26] Adverse reactions of the subject must be followed from the date of giving consent to the subject until 28 days after the last dose of the test drug or the start of subsequent chemotherapy, whichever occurs first. In the event of significant or drug-related toxicity, subjects will be followed until resolved or stabilized
