# Supplementary material for: Lazertinib in EGFR-Variant Non–Small Cell Lung Cancer With CNS Failure to Prior EGFR Tyrosine Kinase Inhibitors: A Nonrandomized Controlled Trial
Source: JAMA Oncol. 2024 Aug 15;10(10):1342–51. doi: 10.1001/jamaoncol.2024.2640 (PMC11327907; doi:10.1001/jamaoncol.2024.2640)
Supplement: Supplement 2. — eTable 1. Cerebrospinal Fluid (CSF) Penetration Rate of Lazertinib and Its Metabolite (YH26334) eTable 2. Overall Summary of Adverse Events (AEs) and Treatment-Related AEs (TRAEs) eTable 3. Incidence of Treatment-Related Adverse Events (TRAEs) eTable 4. Genetic Variant Analysis Based on Blood-Based Next-Generation Sequencing at Baseline and the Time of Progression eFigure 1. Intracranial PFS (iPFS) Based on the Neurologic Symptom, Overall Survival, and Restricted Mean Duration of Response (DoR) eFigure 2. Swimmer Plot for Treatment Duration and Time to Response of (A) Intracranial, (B) Extracranial, and (C) Overall Tumor Lesions eFigure 3. Genomic Alterations in (A) Baseline Samples of All Enrolled Patients (N = 40) and (B) Paired Pre- and Post-Lazertinib Samples in 14 Patients Who Showed Disease Progression by Blood-Based Next-Generation Sequencing (NGS) [file jamaoncol-e242640-s002.pdf]

Supplemental Online Content

Hong MH, Choi YJ, Ahn HK, et al. Lazertinib in *EGFR*-Variant Non–Small Cell Lung Cancer With CNS Failure to Prior EGFR Tyrosine Kinase Inhibitors. *JAMA Oncol*. Published online August 8, 2024. doi:10.1001/jamaoncol.2024.2640

eTable 1. Cerebrospinal Fluid (CSF) Penetration Rate of Lazertinib and Its Metabolite (YH26334)

eTable 2. Overall Summary of Adverse Events (AEs) and Treatment-Related AEs (TRAEs)

eTable 3. Incidence of Treatment-Related Adverse Events (TRAEs)

eTable 4. Genetic Variant Analysis Based on Blood-Based Next-Generation Sequencing at Baseline and the Time of Progression

eFigure 1. Intracranial PFS (iPFS) Based on the Neurologic Symptom, Overall Survival, and Restricted Mean Duration of Response (DoR)

eFigure 2. Swimmer Plot for Treatment Duration and Time to Response of (A) Intracranial, (B) Extracranial, and (C) Overall Tumor Lesions

eFigure 3. Genomic Alterations in (A) Baseline Samples of All Enrolled Patients (N = 40) and (B) Paired Pre- and Post-Lazertinib Samples in 14 Patients Who Showed Disease Progression by Blood-Based Next-Generation Sequencing (NGS)

This supplementary material has been provided by the authors to give readers additional information about their work.

© 2024 Hong MH et al. *JAMA Oncology*.

eTable 1. Cerebrospinal Fluid (CSF) Penetration Rate of Lazertinib and Its Metabolite (YH26334)

	Lazertinib (240mg)	YH26334[a]
No. of samples	6	4
CSF/Free Plasma (%)	46.2	33.1

[a]The active metabolite of YH25448 (YH26334) is present in humans at levels approximately 3% those of the parent (NCT03046992).

© 2024 Hong MH et al. *JAMA Oncology*.

eTable 2. Overall Summary of Adverse Events (AEs) and Treatment-Related AEs (TRAEs)

<table>
<tr><th colspan="2">AEs</th><th>N (%)</th></tr>
<tr><td colspan="2">Any AEs</td><td>39 (97.5)</td></tr>
<tr><td colspan="2">Any TRAEs</td><td>35 (87.5)</td></tr>
<tr><td colspan="2">Any AEs Grade ≥ 3</td><td>15 (37.5)</td></tr>
<tr><td colspan="2">Any TRAEs Grade ≥ 3</td><td>4 (10.0)</td></tr>
<tr><td colspan="2">Any serious AEs</td><td>15 (37.5)</td></tr>
<tr><td colspan="2">Any treatment-related serious AEs</td><td>5 (12.5)</td></tr>
<tr><td colspan="2">Any AEs leading to death</td><td>3 (7.5)</td></tr>
<tr><td colspan="2">Any TRAEs leading to death</td><td>-</td></tr>
<tr><td rowspan="3">Any AEs leading to</td><td>Temporary drug interruption</td><td>14 (35.0)</td></tr>
<tr><td>Dose reduction</td><td>8 (20.0)</td></tr>
<tr><td>Permanent discontinuation</td><td>3 (7.5)</td></tr>
</table>

© 2024 Hong MH et al. *JAMA Oncology*.

eTable 3. Incidence of Treatment-Related Adverse Events (TRAEs)

Adverse events	All grades, n (%)	Grade 3 or more, n (%)
Paresthesia	18 (45.0)	-
Skin rash	17 (42.5)	-
Pruritus	11 (27.5)	-
Diarrhea	8 (20.0)	1 (2.5)
Paronychia	7 (17.5)	-
Stomatitis	7 (17.5)	-
Neuropathy peripheral	5 (12.5)	-
Aspartate aminotransferase increased	5 (12.5)	-
Alanine aminotransferase increased	3 (7.5)	-
Constipation	3 (7.5)	-
Dizziness	3 (7.5)	-
Fatigue	3 (7.5)	1 (2.5)
Nausea	3 (7.5)	-
Asthenia	2 (5.0)	-
Decreased appetite	2 (5.0)	-
Muscle spasms	2 (5.0)	-
Peripheral sensory neuropathy	2 (5.0)	-
Pneumonitis	2 (5.0)	1 (2.5)
Atrial fibrillation	1 (2.5)	-
Blood creatinine increased	1 (2.5)	-
Dry skin	1 (2.5)	-
Headache	1 (2.5)	-
Insomnia	1 (2.5)	-
Muscular weakness	1 (2.5)	-
Myalgia	1 (2.5)	-
Edema	1 (2.5)	-
Edema peripheral	1 (2.5)	-
Palpitations	1 (2.5)	-
Peripheral motor neuropathy	1 (2.5)	-
Platelet count decreased	1 (2.5)	-
Pleural effusion	1 (2.5)	1 (2.5)
Skin atrophy	1 (2.5)	

© 2024 Hong MH et al. *JAMA Oncology.*

eTable 4. Genetic Variant Analysis Based on Blood-Based Next-Generation Sequencing at Baseline and the Time of Progression

ID	Baseline		Best intracranial response	Progressive disease status and type of progression	Progression	
	Detected Alteration(s)	% cfDNA or Amplification			Detected Alteration(s)	% cfDNA or Amplification
SYUH01	Not detected	Not detected	SD	PD (intracranial)	Not detected	Not detected
SYUH02	EGFR E19del	1.2%	SD	Ongoing		
	APC R1114*	1.1%				
SYUH03	EGFR T790M	0.40%	PR	Ongoing		
	EGFR E19del	1.00%				
	APC A1325_Q1328 delinsVS*	0.30%				
SYUH04	Not detected	NOT DETECTED	PR	Ongoing		
SYUH06	Not detected	NOT DETECTED	SD	PD (intracranial)	Not detected	Not detected
SYUH07	EGFR L858R	0.30%	SD	PD (extracranial)	EGFR L858R	6.00%
	PIK3CA V344M	0.70%			PIK3CA V344M	7.90%
					CDK4 Amplification	High (+++)
SYUH08	JAK2 V617F	2.70%	PR	Ongoing		
SYUH09	Not detected	Not detected	SD	PD (intra- & extracranial)	TP53 C242fs	0.30%
SYUH10	EGFR E19del	0.70%	PR	PD (intracranial)		
SYUH11	EGFR T790M	4.60%	PR	PD (extracranial)		
	EGFR L858R	7.70%				
	EGFR Amplification	Low (+)				
	CTNNB1 I35_G38del	1.60%				
SYUH12	Not detected	Not detected	PR	Ongoing		
SYUH13	KRAS V14I	0.10%	SD	PD (intra- & extracranial)	NOT DETECTED	
SYUH14	NOT DETECTED	Not detected	PR	PD (extracranial)	ATM P2222fs	0.10%
SYUH15	JAK2 V617F	3.10%	PR	Ongoing		
SYUH16	Not detected	Not detected	PR	Ongoing		
SYUH18	EGFR L858R	0.03%	NA	Ongoing		

© 2024 Hong MH et al. *JAMA Oncology.*

**SYUH19**	**EGFR L858R**	**0.07%**	**SD**	**Ongoing**		
**SYUH20**	**EGFR L858R**	**0.80%**	**SD**	**PD (intra- & extracranial)**	**EGFR L858R**	**2.60%**
	**EGFR H870R**	**0.60%**			**EGFR H870R**	**2.60%**
**SYUH21**	**EGFR T790M**	**0.70%**	**SD**	**PD (intracranial)**	**PTEN Q17***	**0.20%**
	**EGFR L858R**	**0.80%**				
	**PTEN Q17***	**0.90%**				
	**TP53 S90fs**	**1.10%**				
**SYUH22**	**EGFR E19del**	**54.10%**	**NA**	**Ongoing**		
	**TP53 E286K**	**61.10%**				
	**PTEN T319fs**	**68.40%**				
	**EGFR Amplification**	**High (+++)**				
	**PIK3CA Amplification**	**High (+++)**				
	**CDH1 R63***	**0.10%**				
	**RB1 C853fs**	**46.50%**				
**SYUH23**	**Not detected**	**Not detected**	**PR**	**Ongoing**		
**SYUH24**	**TP53 P152Q**	**0.20%**	**PR**	**Ongoing**		
**SYUH26**	**Not detected**	**Not detected**	**SD**	**PD (extracranial)**	**EGFR L858R**	**4.00%**
					**TP53 Y163C**	**1.90%**
**SSNU01**	**Not detected**	**Not detected**	**SD**	**Ongoing**		
**SSNU02**	**Not detected**	**Not detected**	**PR**	**Ongoing**		
**SSNU03**	**KRAS T50I**	**0.20%**	**CR**	**Ongoing**		
**SSNU04**	**Not detected**	**Not detected**	**CR**	**PD (intra- & extracranial)**	**E19del**	**0.9%**
					**TP53 S215N**	**0.4%**
					**TP53 V272M**	**0.20%**
**SSNU05**	**Not detected**	**Not detected**	**PR**	**Ongoing**		
**SBSH01**	**TP53D61fs**	**0.03%**	**SD**	**Ongoing**		
**SCUS01**	**EGFR E19del**	**0.10%**	**PR**	**Ongoing**		
**SCUS02**	**ATM R337H**	**0.30%**	**PR**	**Ongoing**		
**SCUS03**	**EGFR E19del**	**1.80%**	**PD**	**Ongoing**		
	**NRAS G12D**	**0.04%**				
	**PIK3CA Amplification**	**High (+++)**				
	**EGFR Amplification**	**Medium (++)**				

© 2024 Hong MH et al. *JAMA Oncology*.

**SCUS04**	**FGFR2 A553T**	**0.30%**	**CR**	**PD (extracranial)**	**E19del**	**0.10%**
**SKAA01**	**EGFR L858R**	**3.50%**	**SD**	**Ongoing**		
	**ATM V1600fs**	**1.20%**				
	**TP53 L194R**	**0.80%**				
**SGGH01**	**EGFR T790M**	**0.20%**	**PR**	**PD (intracranial)**	**EGFR L858R**	**0.50%**
	**EGFR L858R**	**0.60%**			**ATM G2891D**	**0.30%**
	**ATM G2891D**	**0.40%**			**FGFR3-TACC3 Fusion**	**0.07%**
	**TP53 P177H**	**0.40%**			**TP53 P177H**	**0.30%**
	**TP53 V143M**	**0.10%**			**KRAS Amplification**	**Medium (++)**
**SGGH05**	**GNAS E209D**	**0.70%**	**SD**	**Ongoing**		
	**CCNE1 I96F**	**0.10%**				
**SGGH06**	**Not detected**	**Not detected**	**SD**	**PD (extracranial)**	**EGFR L858R**	**0.20%**
					**ATM S1455R**	**26.50%**
					**TP53 Splice Site SNV**	**0.10%**
**SGGH07**	**EGFR L858R**	**0.70%**	**SD**	**Ongoing**		
	**TP53 P278fs**	**0.70%**				
	**APC Q1447***	**0.60%**				
**SGGH08**	**MET I1053T**	**0.20%**	**PR**	**Ongoing**		
**SGGH10**	**EGFR T790M**	**0.80%**	**PR**	**PD (extracranial)**	**E19del**	**0.90%**
	**EGFR E19del**	**1.50%**			**TP53 P278A**	**0.70%**
	**TP53 P278A**	**0.50%**				

CR, complete response; NA, not available; PD, progressive disease; PR, partial response; SD, stable disease

© 2024 Hong MH et al. *JAMA Oncology*.

eFigure 1. Intracranial PFS (iPFS) Based on the Neurologic Symptom, Overall Survival, and Restricted Mean Duration of Response (DoR)

(A) iPFS based on the neurological symptoms, (B) Overall survival, (C) Restricted Mean Intracranial DoR (iDoR), and (D) Restricted Mean DoR.

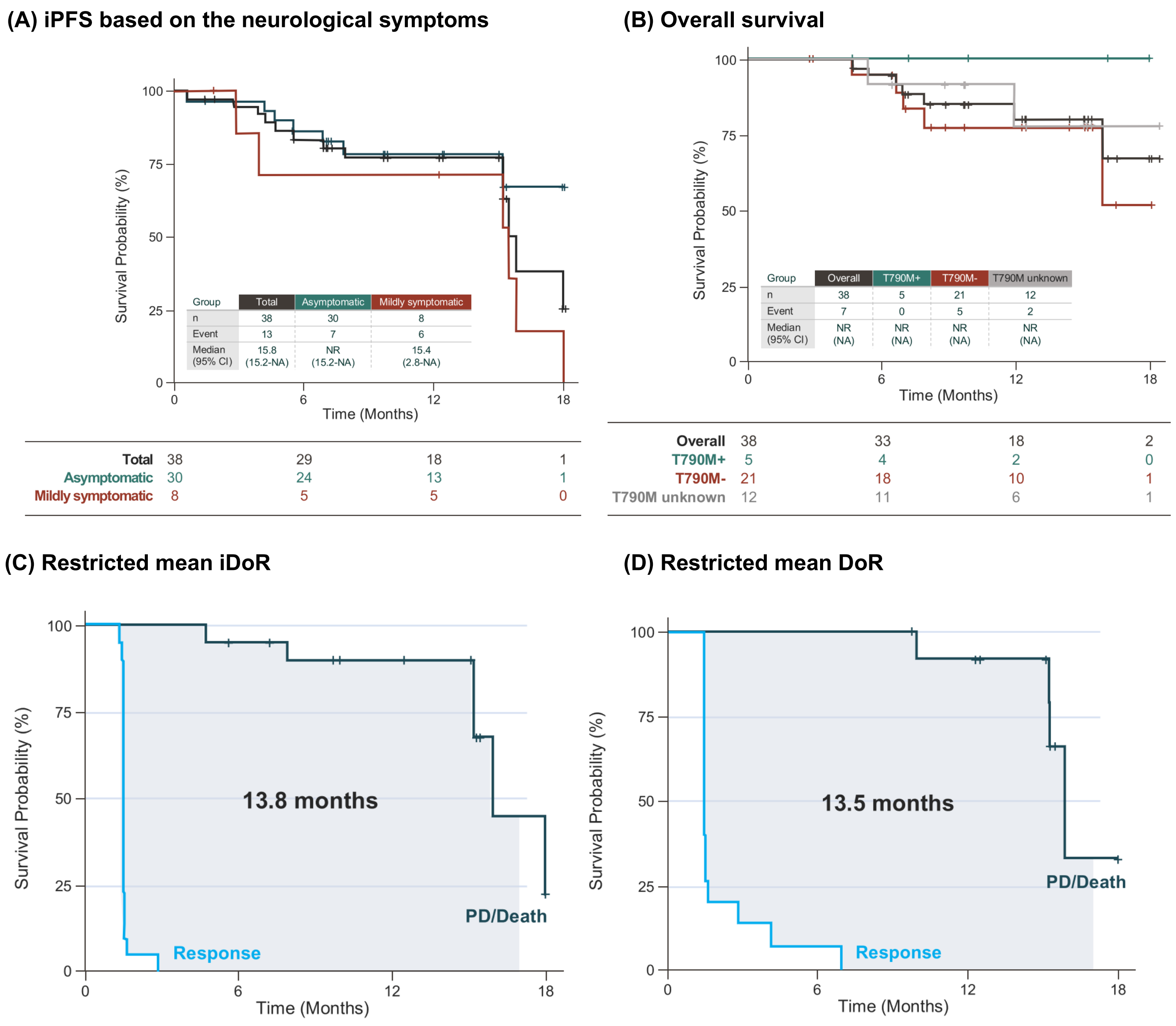

© 2024 Hong MH et al. *JAMA Oncology.*

eFigure 2. Swimmer Plot for Treatment Duration and Time to Response of (A) Intracranial, (B) Extracranial, and (C) Overall Tumor Lesions

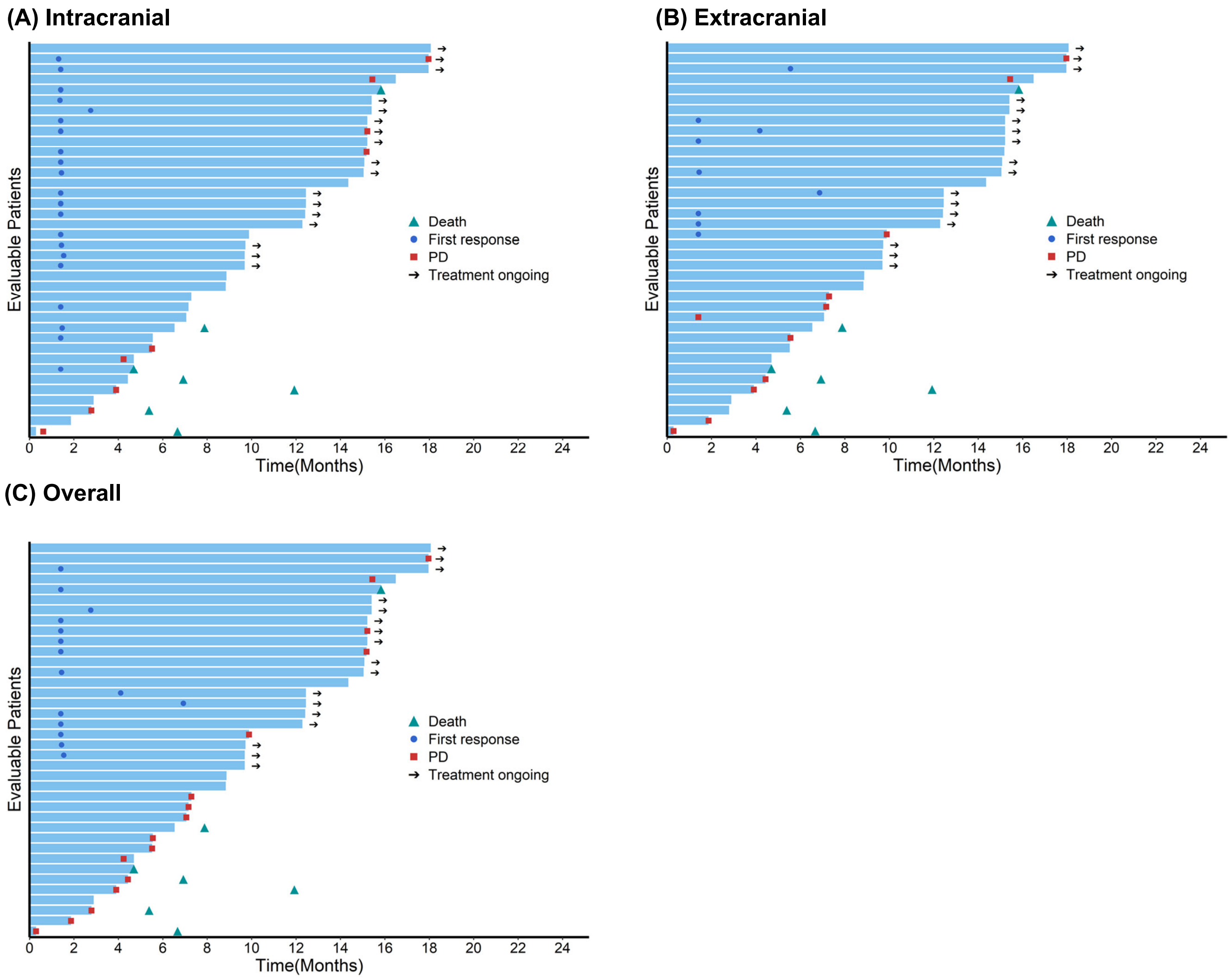

PD, progressive disease

© 2024 Hong MH et al. *JAMA Oncology.*

eFigure 3. Genomic Alterations in (A) Baseline Samples of All Enrolled Patients (N = 40) and (B) Paired Pre- and Post-Lazertinib Samples in 14 Patients Who Showed Disease Progression by Blood-Based Next-Generation Sequencing (NGS)

(A) Baseline

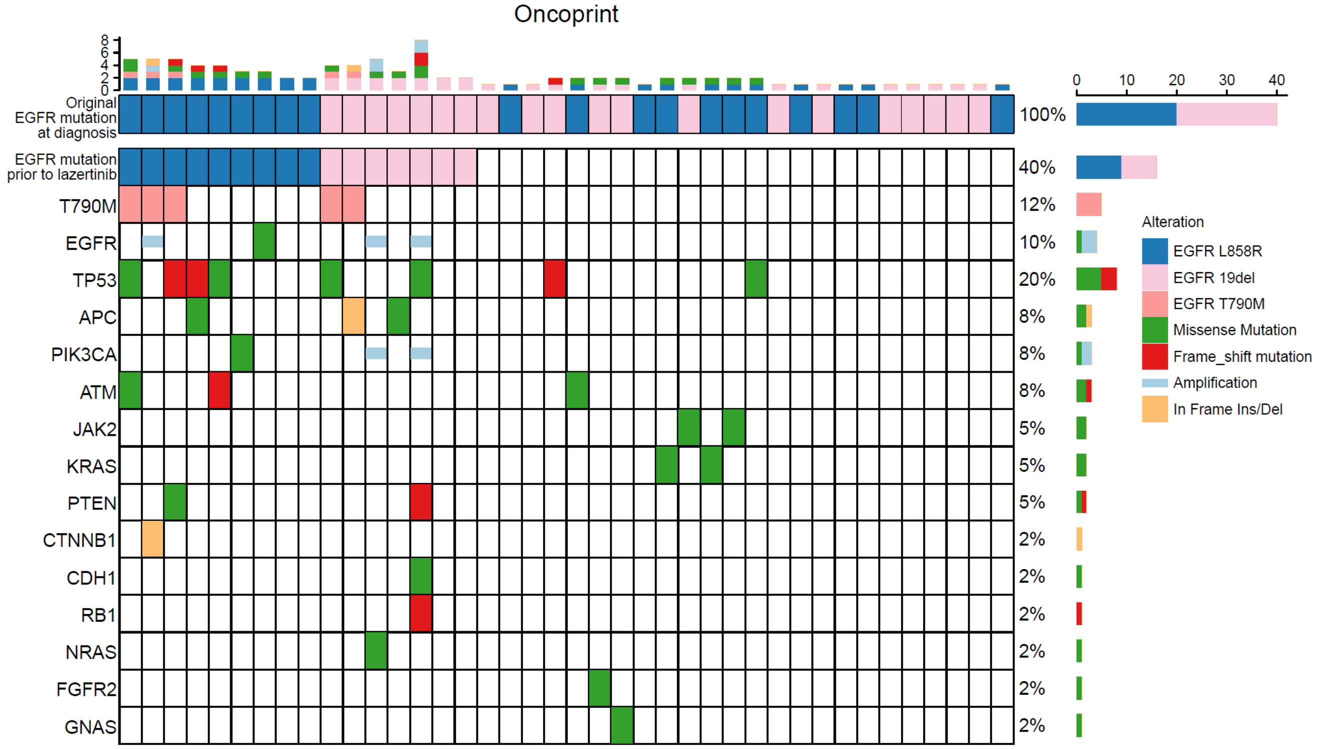

(B) Pre-lazertinib and post-lazertinib

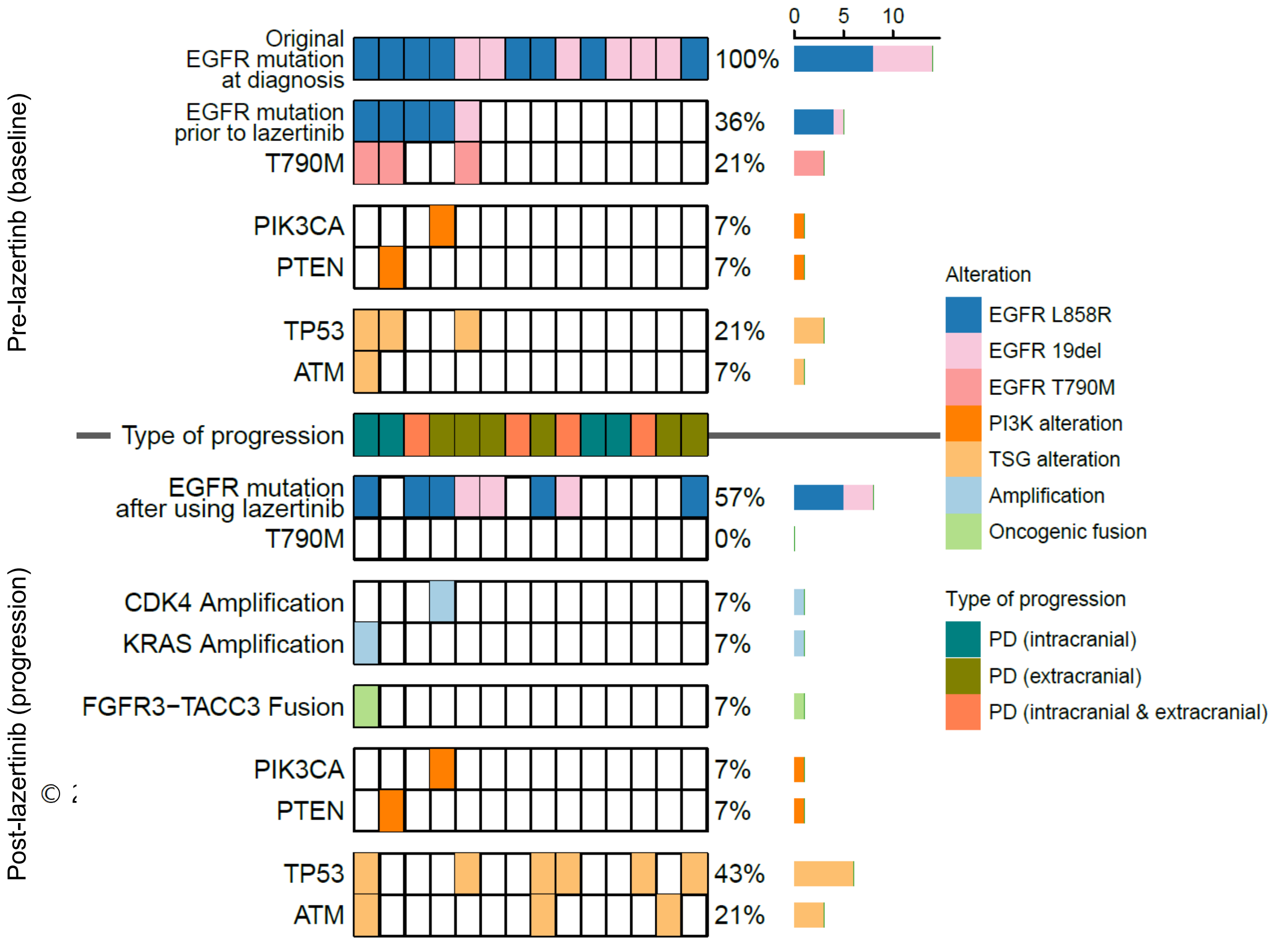

© 2024 Hong MH et al. *JAMA Oncology*.
