# Supplementary material for: Lazertinib in EGFR-Variant Non–Small Cell Lung Cancer With CNS Failure to Prior EGFR Tyrosine Kinase Inhibitors: A Nonrandomized Controlled Trial
Source: JAMA Oncol. 2024 Aug 15;10(10):1342–51. doi: 10.1001/jamaoncol.2024.2640 (PMC11327907; doi:10.1001/jamaoncol.2024.2640)
Supplement: Supplement 3. — Data Sharing Statement [file jamaoncol-e242640-s003.pdf]

Data Sharing Statement

Hong. Lazertinib in EGFR-Variant Non–Small Cell Lung Cancer With CNS Failure to Prior EGFR Tyrosine Kinase Inhibitors. *JAMA Oncol*. Published August 08, 2024. doi:10.1001/jamaoncol.2024.2640

Data
Data available: Yes
Data types: Deidentified participant data
How to access data: Data available: Yes Data types: Deidentified subject data How to access data: nobleg@yuhs.ac Please email corresponding author for data regarding this clinical trial, data will be released in compliance with institutional policy. When available: With publication
When available: With publication

Supporting Documents
Document types: Other (please specify)
Additional Information: Trial protocol, including the statistical analysis plan, and any amendments
How to access documents: Correspending author, Hye Ryun Kim, email: nobelg@yuhs.ac
When available: With publication

Additional Information
Who can access the data: Correspending author, Hye Ryun Kim, email: nobelg@yuhs.ac
Types of analyses: The data will be made available for meta-analyses, provided that researchers adhere to the data use agreements and ethical guidelines stipulated by the institutions which enrolled paritipants.
Mechanisms of data availability: After approval of a proposal
